# Supplementary material for: Group-Level Progressive Alterations in Brain Connectivity Patterns Revealed by Diffusion-Tensor Brain Networks across Severity Stages in Alzheimer’s Disease
Source: Front Aging Neurosci. 2017 Jul 7;9:215. doi: 10.3389/fnagi.2017.00215 (PMC5500648; doi:10.3389/fnagi.2017.00215)
Supplement: Supplementary file 1 [file Table_1.DOCX]

Table S1: ADNI subjects within each group

EMCI: Early mild cognitive impairment; LMCI=Late mild cognitive impairment; AD= Alzheimer disease; M=Male, F=Female.

| **Control** | | | **EMCI** | | | | **LMCI** | | | | **AD** | | |
| --- | --- | --- | --- | --- | --- | --- | --- | --- | --- | --- | --- | --- | --- |
| *SubjectId* | *Sex* | *Age* | *SubjectId* | *Sex* | *Age* | *SubjectId* | | *Sex* | *Age* | *SubjectId* | | *Sex* | *Age* |
| 003_S_4119 | M | 79 | 003_S_2374 | F | 81 | 003_S_0908 | | F | 70 | 003_S_4373 | | F | 71 |
| 003_S_4839 | M | 66 | 007_S_2394 | M | 69 | 003_S_4354 | | M | 76 | 003_S_5165 | | M | 79 |
| 007_S_4488 | M | 73 | 016_S_4575 | F | 62 | 016_S_4584 | | F | 78 | 003_S_5187 | | F | 62 |
| 007_S_4516 | M | 72 | 021_S_2077 | M | 81 | 016_S_4646 | | F | 61 | 005_S_4707 | | M | 68 |
| 007_S_4620 | M | 77 | 021_S_2100 | F | 88 | 016_S_4902 | | F | 75 | 005_S_4910 | | F | 82 |
| 016_S_4121 | M | 89 | 021_S_2125 | F | 78 | 021_S_4402 | | F | 73 | 005_S_5038 | | M | 82 |
| 021_S_4558 | F | 71 | 021_S_2142 | F | 83 | 021_S_4633 | | F | 73 | 005_S_5119 | | F | 77 |
| 029_S_4279 | M | 84 | 021_S_4419 | F | 65 | 021_S_4857 | | M | 68 | 007_S_4568 | | F | 71 |
| 029_S_4290 | M | 74 | 021_S_4659 | M | 86 | 027_S_4729 | | F | 78 | 007_S_4911 | | M | 75 |
| 029_S_4384 | M | 62 | 021_S_4744 | F | 73 | 027_S_4757 | | F | 63 | 007_S_5196 | | F | 73 |
| 029_S_4385 | F | 68 | 029_S_2370 | F | 64 | 027_S_4804 | | M | 80 | 016_S_4591 | | F | 66 |
| 029_S_4585 | M | 66 | 029_S_2395 | M | 73 | 027_S_4869 | | M | 77 | 016_S_4887 | | M | 75 |
| 029_S_4652 | M | 79 | 029_S_4327 | M | 83 | 027_S_4873 | | M | 83 | 016_S_4963 | | F | 72 |
| 057_S_0934 | F | 77 | 029_S_5135 | M | 77 | 027_S_4936 | | M | 78 | 016_S_5057 | | M | 75 |
| 094_S_4234 | M | 70 | 094_S_2201 | F | 64 | 027_S_4943 | | M | 76 | 016_S_5251 | | F | 66 |
| 094_S_4459 | F | 68 | 094_S_2216 | M | 69 | 027_S_4955 | | M | 72 | 021_S_4718 | | M | 79 |
| 094_S_4460 | F | 67 | 094_S_2238 | M | 69 | 052_S_4626 | | M | 69 | 021_S_4924 | | M | 77 |
| 094_S_4503 | F | 72 | 094_S_2367 | M | 75 | 052_S_4807 | | F | 72 | 027_S_4801 | | M | 78 |
| 094_S_4649 | M | 66 | 094_S_4434 | M | 68 | 052_S_4945 | | M | 57 | 027_S_4802 | | M | 83 |
| 098_S_4002 | F | 74 | 098_S_2052 | M | 74 | 057_S_4888 | | M | 75 | 027_S_4938 | | M | 71 |
| 098_S_4003 | F | 72 | 098_S_2071 | M | 85 | 057_S_4909 | | F | 78 | 027_S_4962 | | F | 80 |
| 098_S_4018 | M | 76 | 099_S_4205 | F | 84 | 094_S_4162 | | F | 71 | 027_S_4964 | | M | 81 |
| 098_S_4050 | M | 77 | 099_S_4498 | F | 80 | 094_S_4295 | | F | 70 | 052_S_5062 | | F | 71 |
| 098_S_4275 | M | 73 | 109_S_2110 | F | 68 | 094_S_4630 | | F | 66 | 094_S_4089 | | M | 74 |
| 098_S_4506 | M | 72 | 109_S_2111 | M | 72. | 109_S_4471 | | M | 73 | 094_S_4737 | | F | 74 |
| 099_S_4076 | F | 75 | 109_S_2200 | F | 76 | 109_S_4531 | | M | 74 | 098_S_4201 | | F | 64 |
| 127_S_4148 | M | 73 | 109_S_4380 | M | 72 | 126_S_4458 | | F | 76 | 098_S_4215 | | M | 82 |
| 127_S_4198 | F | 78 | 109_S_4455 | M | 64 | 126_S_4507 | | M | 78 | 109_S_4378 | | M | 80 |
| 127_S_4604 | M | 65 | 109_S_4594 | M | 62 | 126_S_4675 | | M | 80 | 126_S_4494 | | M | 71 |
| 127_S_4645 | F | 76 | 126_S_2360 | M | 64 | 126_S_4712 | | M | 74 | 127_S_4749 | | F | 78 |
| 127_S_4843 | F | 73 | 126_S_4891 | M | 60 | 126_S_4743 | | M | 70 | 127_S_4992 | | F | 64 |
| 129_S_0778 | M | 80 | 127_S_4301 | M | 75 | 126_S_4896 | | M | 68 | 127_S_5028 | | M | 62 |
| 129_S_4369 | M | 70 | 127_S_4624 | F | 78 | 127_S_4197 | | M | 79 | 127_S_5056 | | M | 85 |
| 129_S_4371 | M | 70 | 127_S_4765 | M | 76 | 127_S_4210 | | M | 64 | 127_S_5058 | | M | 62 |
| 129_S_4396 | F | 81 | 129_S_2347 | M | 73 | 127_S_4240 | | M | 71 | 127_S_5067 | | M | 81 |
| 131_S_0123 | M | 81 | 129_S_4220 | F | 73 | 129_S_4287 | | F | 73 | 127_S_5095 | | M | 66 |
