# Supplementary material for: Group-Level Progressive Alterations in Brain Connectivity Patterns Revealed by Diffusion-Tensor Brain Networks across Severity Stages in Alzheimer’s Disease
Source: Front Aging Neurosci. 2017 Jul 7;9:215. doi: 10.3389/fnagi.2017.00215 (PMC5500648; doi:10.3389/fnagi.2017.00215)
Supplement: Supplementary file 2 [file Table_2.docx]

Table S2: Examples of pseudo F-statistics, between-group and within-group sum of squares. Three different situations: Node 10, that does not provide any significant change in pattern connectivity; Node 16, significantly different in stage III; and Node 18, with pattern connectivity significantly different in stages II and III.

EMCI: Early mild cognitive impairment; LMCI=Late mild cognitive impairment; AD= Alzheimer disease

| **Module** | **Control vs EMCI** | | | | **Control vs LMCI** | | | | **Control vs AD** | | |
| --- | --- | --- | --- | --- | --- | --- | --- | --- | --- | --- | --- |
|  | *F* | *SSA* | *SSW* | *F* | | *SSA* | *SSW* | *F* | | *SSA* | *SSW* |
| 10 | 0.527 | 0.008 | 1.028 | 0.630 | | 0.008 | 0.940 | 0.894 | | 0.012 | 0.941 |
| 16 | 0.380 | 0.019 | 3.457 | 0.285 | | 0.014 | 3.404 | 3.410 | | 0.173 | 3.550 |
| 18 | 3.057 | 0.024 | 0.558 | 5.854 | | 0.049 | 0.595 | 6.018 | | 0.051 | 0.588 |
