# Supplementary material for: Group-Level Progressive Alterations in Brain Connectivity Patterns Revealed by Diffusion-Tensor Brain Networks across Severity Stages in Alzheimer’s Disease
Source: Front Aging Neurosci. 2017 Jul 7;9:215. doi: 10.3389/fnagi.2017.00215 (PMC5500648; doi:10.3389/fnagi.2017.00215)
Supplement: Supplementary file 3 [file Table_3.docx]

Table S3: Group comparison not involving the healthy control group.

EMCI: Early mild cognitive impairment; LMCI=Late mild cognitive impairment; AD= Alzheimer disease * 0.01<*p*<0.05; ** 0.005 < *p* <0.01: *** *p*<0.005.

| **Module** | **EMCI vs LMCI** | **LMCI vs AD** | **EMCI vs AD** |
| --- | --- | --- | --- |
| 1 | 0.869 | 0.089 | 0.153 |
| 2 | 0.473 | 0.089 | 0.049* |
| 3 | 0.474 | 0.089 | 0.049* |
| 4 | 0.473 | 0.433 | 0.049* |
| 5 | 0.474 | 0.433 | 0.352 |
| 6 | 0.736 | 0.372 | 0.969 |
| 7 | 0.869 | 0.395 | 0.688 |
| 8 | 0.869 | 0.395 | 0.055 |
| 9 | 0.473 | 0.235 | 0.352 |
| 10 | 0. 473 | 0.222 | 0.383 |
| 11 | 0. 869 | 0.533 | 0.969 |
| 12 | 0. 473 | 0.395 | 0.383 |
| 13 | 0.473 | 0.089 | 0.352 |
| 14 | 0.473 | 0.060 | 0.035* |
| 15 | 0.930 | 0.089 | 0.092 |
| 16 | 0.869 | 0.089 | 0.049* |
| 17 | 0.869 | 0.410 | 0.905 |
| 18 | 0.736 | 0.698 | 0.623 |
| 19 | 0.474 | 0.089 | 0.383 |
| 20 | 0.787 | 0.698 | 0.969 |
